# Hidden Treasures: Microbial Natural Product Biosynthesis off the Beaten Path

**DOI:** 10.1128/msystems.00846-21

**Published:** 2021-08-31

**Authors:** Friederike Biermann, Eric J. N. Helfrich

**Affiliations:** a Institute for Molecular Bio Science, Goethe University Frankfurt, Frankfurt, Germany; b LOEWE Center for Translational Biodiversity Genomics (TBG), Frankfurt, Germany

**Keywords:** bioinformatics, biosynthesis, biosynthetic gene cluster, genome mining, natural products, peptides, polyketides, terpenes

## Abstract

Microbes produce structurally diverse natural products to interact with their environment. Many of the biosynthetic products involved in this “metabolic small talk” have been exploited for the treatment of various diseases. As an alternative to the traditional bioactivity-guided workflow, genome mining has been introduced for targeted natural product discovery based on genome sequence information. In this commentary, we will discuss the evolution of genome mining, as well as its current limitations. The Helfrich laboratory aims to play a leading role in overcoming these limitations with the development of computational strategies to identify noncanonical biosynthetic pathways and to decipher the principles that govern the production of the associated metabolites. We will use these insights to develop algorithms for the prediction of natural product scaffolds. These studies will pave the way toward a more comprehensive understanding of the full biosynthetic repertoire encoded in microbial genomes and provide access to novel metabolites.

## COMMENTARY

Natural products, traditionally isolated by bioactivity-guided fractionation, have been exploited as drugs, agrochemicals, and additives in the food and cosmetic industry, and have served as inspiration for many generations of chemists. In recent years, genome mining has revolutionized microbial natural product discovery. Driven by the advancements in genome sequencing technologies, the pool of microbial sequence information is increasing at a rapid pace. Genes responsible for the biosynthesis of a natural product are typically clustered in microbial genomes. Biosynthetic studies on model natural product pathways resulted in the identification of universal biosynthetic principles for the most commonly studied classes of natural products (1–3). Even though the biosynthetic products within a natural products class are highly diverse, their production follows common reactions catalyzed by core biosynthetic enzymes. The genes encoding these enzymes exhibit a certain degree of homology based on which they can be identified in genome sequences. Genome mining, i.e., the screening of genome sequences for their natural product biosynthetic potential, has revealed that we have vastly underestimated the biosynthetic repertoire of microbes as previously extrapolated from traditional bioactivity-guided fractionation efforts (4). These revelations have resulted in the development of more and more sophisticated bioinformatic platforms for the identification, annotation, prioritization, and massive parallel analysis of natural product biosynthetic gene clusters (BGCs).

## THE EVOLUTION OF GENOME MINING

An excellent example for the evolution of genome mining is *trans*-acyl transferase polyketide synthases (*trans*-AT PKSs). *Trans*-AT PKSs are arguably the most complex bacterial natural product assembly lines known today. They are responsible for the production of a wealth of structurally diverse polyketides with a remarkable array of bioactivities. *Trans*-AT PKSs form giant enzymatic assembly lines that can be subdivided into individual modules that are responsible for the incorporation of a building block and/or the modification to the growing polyketide chain (5). Due to their overall architectural similarity to textbook polyketide synthases (*cis*-AT PKSs), genome mining pipelines were capable of identifying *trans*-AT PKS BGCs from early on, even though their biosynthetic blueprints were poorly understood (6). This lack of biosynthetic understanding can be attributed to numerous enzymatic peculiarities in the *trans*-AT PKS systems (5, 7). Therefore, a new set of biosynthetic rules that govern *trans*-AT PKS biosynthesis had to be established (8). It took until 2008 for the biosynthetic blueprints of *trans*-AT PKSs to be deciphered (9). Due to the small data set of characterized *trans*-AT PKS BGCs at the time, the resolution of the predicted monomers was still relatively low and resulted in a certain level of uncertainty in the predicted polyketide structures (9). In addition, the manual prediction of *trans*-AT PKS products was time-consuming and required a well-curated data set and extensive phylogenetic analysis.

**FIG 1:**
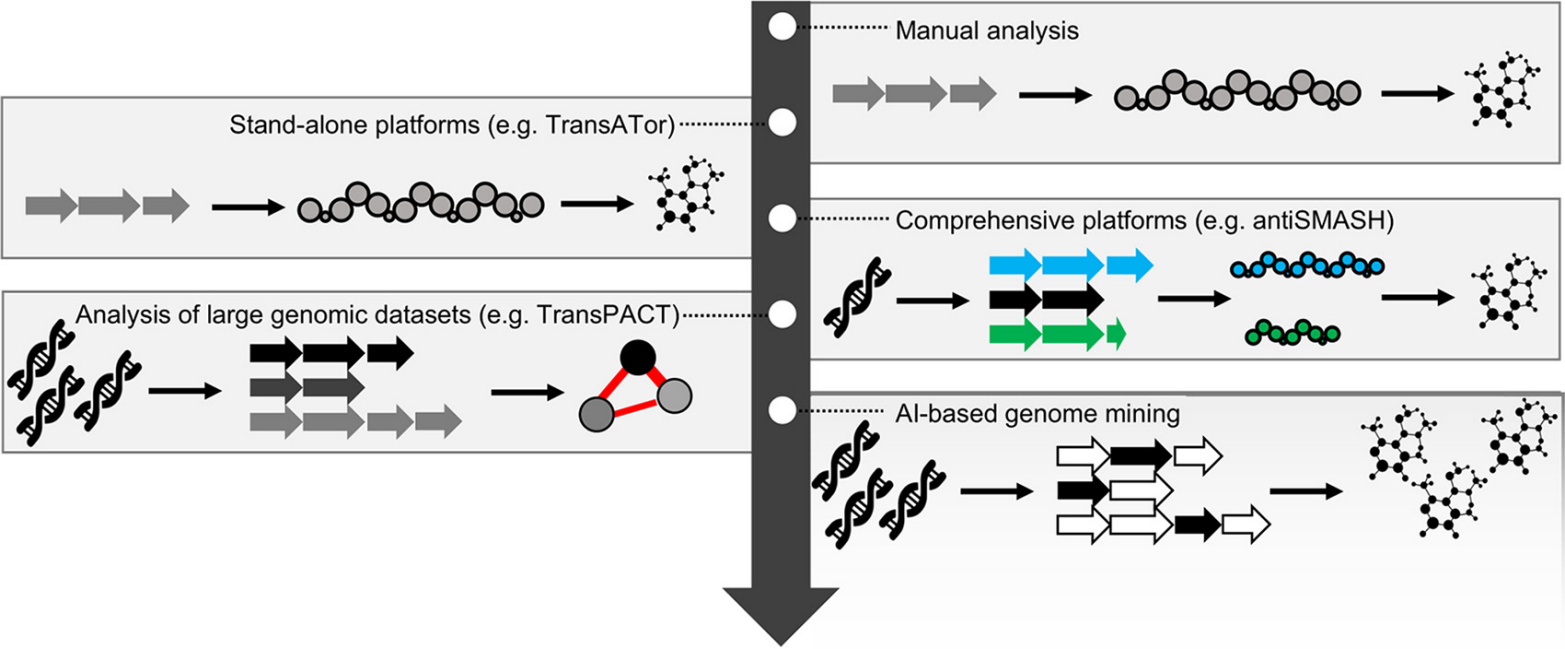
The evolution of genome mining. Arrows in different shades of gray represent genes of the same natural product class. Different colors indicate different natural product classes. White arrows represent noncanonical genes that have evolved to fulfill the function of textbook core biosynthetic genes. The fading background in the AI-based genome mining box that is open toward the present and future indicates its importance for the development of novel genome mining tools.

The integration of extensive biochemical (10), knockout (11), and bioinformatic studies (8) resulted in a significantly improved biosynthetic understanding. These deeper biosynthetic insights were used for the development of transATor, an algorithm for the automated prediction of *trans*-AT PKS-derived polyketide core structures (8) (Fig. 1). The predicted structures can be used for *in silico* dereplication studies, to prioritize *trans*-AT PKS BGCs, and to guide the isolation and structure elucidation process (8). To study the origins of chemical diversity encoded in the blueprints of all sequenced *trans*-AT PKS systems, the *trans*-AT PKS Annotation and Comparison Tool (TransPACT) was subsequently developed in close collaboration with the Medema and Piel groups for the massive parallel analysis of all *trans*-AT PKS BGCs identified in genomes deposited in GenBank (12). The analysis of more than 1,700 BGCs revealed that *trans*-AT PKSs evolve through extensive recombination of conserved arrangements of consecutive module series (12). In addition to these evolutionary insights, TransPACT can be used to probe the biosynthetic space of *trans*-AT PKSs and to prioritize and dereplicate *trans*-AT PKS BGCs (12). TransPACT and features of transATor are currently being integrated into antiSMASH, the most comprehensive and widely used genome mining pipeline (13), to visualize evolutionary relationships between *trans*-AT PKS BGCs and predict the structures of the associated metabolites.

## POSSIBILITIES AND LIMITATIONS OF CURRENT GENOME MINING PIPELINES

**FIG 2:**
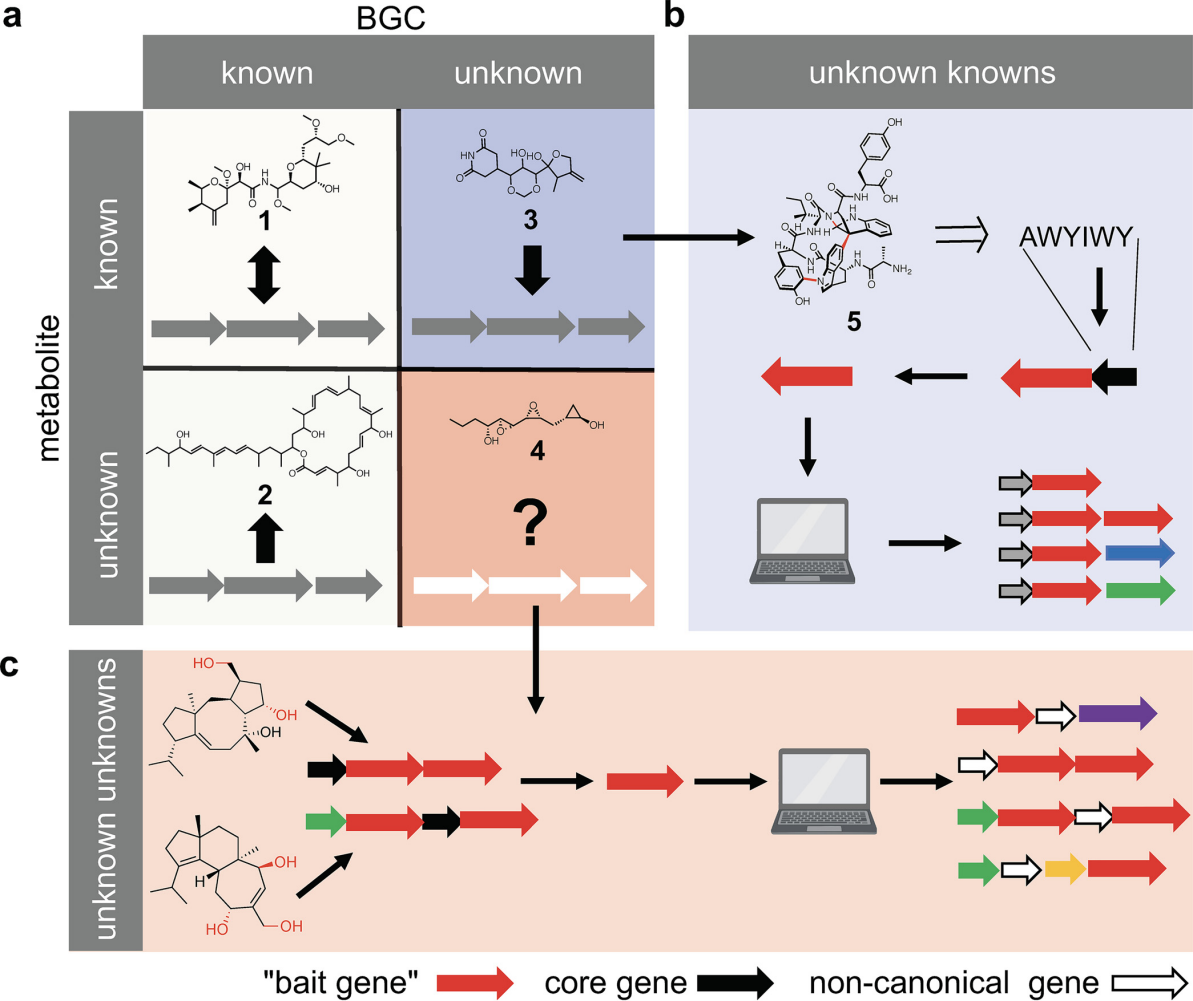
Biosynthetic gene cluster/natural product pairs can be grouped into four categories. Concepts for establishing novel BGC-metabolite links for the unknown knowns and unknown unknowns are depicted in b and c, respectively. Core biosynthetic genes are depicted as black arrows, tailoring genes used as bait are shown in red, and other tailoring genes are shown in blue, green, purple, and orange. White arrows represent noncanonical genes that have evolved to fulfill the function of textbook core biosynthetic genes.

State-of-the-art genome mining algorithms, as exemplified by the *trans*-AT PKS genome mining tools, show great potential for the identification, dereplication, and prioritization of canonical biosynthetic pathways. They are, however, limited in the sense that they frequently overlook pathways that differ significantly from the seemingly universal biosynthetic principles established for the different natural product classes. BGC-metabolite pairs can be conceptually grouped into four categories (Fig. 2a) as follows: (i) known metabolite and known BCG; (ii) known metabolite and unknown BGC; (iii) unknown metabolite and known BGC; and (iv) unknown metabolite and unknown BGC. Only two of the groups are reliably covered by current genome mining pipelines that are based on hard-coded biosynthetic rules: First, there are the known BGCs that are linked to known metabolites (known knowns). Recent examples include the characterization of new members of the spliceostatin family of splicing inhibitors from a plant pathogen (12) or the identification of a cultured source of a pederin-type polyketide [1] (14). The second category represents the classical genome mining workflow, i.e., the targeted identification of a metabolite that is associated with an orphan BGC (known unknowns). A recent example includes the identification of the narrow-spectrum antibiotic macrobrevin [2] from a member of the Arabidopsis thaliana leaf microbiome (15). Furthermore, there are cases in which a metabolite is characterized but the corresponding BGC has not been identified (unknown known). Sesbanimide [3] is a recent example of a small *trans*-AT PKS-derived antitumor compound that was initially isolated from Sesbania drummondii plant seeds almost 40 years ago (16) and that was only recently identified as a truncated, bacterial polyketide biosynthesized by a large *trans*-AT PKS (17). The fourth category comprises the real hidden biosynthetic treasures: noncanonical biosynthetic pathways that are currently overlooked by state-of-the-art bioinformatic platforms. Categorized as the unknown unknowns, neither the BGC is identified by profile hidden Markovm-based approaches, nor is the corresponding compound characterized. One recent example from the world of *trans*-AT PKSs is toblerol [4] (18). Toblerol is the product of an unusual *trans*-AT PKS-like assembly line that was overlooked even by the most sophisticated bioinformatic platforms because it follows as-of-yet poorly understood biosynthetic principles that are “somewhat off the beaten *trans*-AT PKS biosynthetic path” (18). To open the treasure chest of these noncanonical biosynthetic pathways, machine learning-based approaches are currently being developed to identify BGCs based on pattern recognition rather than hard-coded biosynthetic principles.

## SYSTEMATIC IDENTIFICATION OF NONCANONICAL BIOSYNTHETIC PATHWAYS

Motivated by the accidental encounter of multiple category three and four BGCs, we have recently started wandering off the beaten biosynthetic tracks in pursuit of the targeted and systematic identification of biosynthetic pathways that do not follow canonical biosynthetic principles. To do so, we are employing two conceptually orthologous strategies. In many cases, unprecedented metabolites that do not seem to be biosynthesized by canonical pathways have been reported in the literature. The first step toward identifying the associated BGC is the retrobiosynthetic analysis of the metabolite of interest. This analysis usually results in one or multiple alternative biosynthetic hypotheses that are subsequently validated in the laboratory. The insights gained from these studies are then employed for the development of machine learning-based algorithms for the identification of BGCs that employ similar biosynthetic principles. One example from our own research deals with the unusual family of atropopeptides that are characterized by complex and highly rigid three-dimensional shapes (19). First annotated as a nonribosomally synthesized peptide (20), we have recently identified tryptorubin A [5], the first representative of the atropopeptides, as a product of a ribosomally synthesized and posttranslationally modified peptide (RiPP) BGC (19). We have developed a machine learning-based algorithm that uses the characteristic atropopeptide tailoring enzyme as bait for the discovery of distantly related RiPP BGCs that encode similar tailoring enzymes and to predict the core structures of the associated peptides (Fig. 2b) (Biermann et al., unpublished). Similar bait-based approaches have been developed for other RiPP families (e.g., RODEO [21] and RiPPER [22]).

An orthologous, structure-independent approach involves the identification of alternative biosynthetic strategies for conserved key reactions within a natural product class. These include BGCs in which seemingly essential biosynthetic genes that are used for the identification of a biosynthetic pathway are replaced by genes of other gene families that have evolved to fulfill said function. A recent example for this hypothesis-driven approach from our laboratory includes the machine learning-based discovery of noncanonical terpene BGCs (Fig. 2c; Biermann et al., unpublished). Terpene biosynthesis can be conceptually divided into two phases. First, isoprene units are polymerized to a linear oligoprenyl precursor that is then transformed into one or multiple complex hydrocarbon backbones by a terpene cyclase. In a second phase, the hydrocarbon backbone is heavily modified by tailoring enzymes to yield a variety of different terpenes (23). The enzymes responsible for these tailoring reactions can be used as bait in a machine learning-based approach to identify noncanonical terpene BGCs that do not harbor the seemingly essential hydrocarbon scaffold-forming cyclases. Instead, the associated noncanonical terpenes seem to be the products of alternative strategies for the cyclization of the oligoprenyl precursor into complex terpene scaffolds. In comparison to platforms such as DeepBGC (24) and ClusterFinder (25), which identify a wide range of putative noncanonical biosynthetic pathways across all natural product classes, our hypothesis-driven approach is optimized for a subpopulation of the global biosynthetic diversity. This focus results in the more comprehensive characterization of the targeted biosynthetic space with higher confidence and can easily be adapted to similar pattern-based and biosynthetic principle-independent approaches for the targeted identification of noncanonical BGCs associated with other natural product classes.

## OUTLOOK

Genome mining has evolved from manual analyses of genome sequences over stand-alone tools for single natural product classes and comprehensive pipelines for the analysis of single genome sequences toward programs for the comparative analysis of massive data sets comprising hundreds or thousands of genome sequences (Fig. 1). Only recently, AI-based technologies have been introduced into genome mining where they serve to overcome current limitations when it comes to the identification of overlooked biosynthetic pathways or the deciphering of biosynthetic principles that can be used for the structure prediction of natural product scaffolds. These developments will not only pave the way toward a more comprehensive understanding of the full biosynthetic repertoire encoded in microbial genome sequences, but will likely also result in the discovery of novel bioactive metabolites and the identification of unprecedented biochemical transformation that can be exploited or mimicked by organic chemists.

## References

[1] Singh M, Chaudhary S, Sareen D. 2017. Non-ribosomal peptide synthetases: Identifying the cryptic gene clusters and decoding the natural product. J Biosci 42:175–187. doi:10.1007/s12038-017-9663-z. 28229977

[2] Hertweck C. 2009. The biosynthetic logic of polyketide diversity. Angew Chem Int Ed Engl 48:4688–4716. doi:10.1002/anie.200806121. 19514004

[3] Montalban-Lopez M, Scott TA, Ramesh S, Rahman IR, van Heel AJ, Viel JH, Bandarian V, Dittmann E, Genilloud O, Goto Y, Grande Burgos MJ, Hill C, Kim S, Koehnke J, Latham JA, Link AJ, Martinez B, Nair SK, Nicolet Y, Rebuffat S, Sahl HG, Sareen D, Schmidt EW, Schmitt L, Severinov K, Sussmuth RD, Truman AW, Wang H, Weng JK, van Wezel GP, Zhang Q, Zhong J, Piel J, Mitchell DA, Kuipers OP, van der Donk WA. 2021. New developments in RiPP discovery, enzymology and engineering. Nat Prod Rep 38:130–239. doi:10.1039/d0np00027b. 32935693 PMC7864896

[4] Ziemert N, Alanjary M, Weber T. 2016. The evolution of genome mining in microbes—a review. Nat Prod Rep 33:988–1005. doi:10.1039/c6np00025h. 27272205

[5] Helfrich EJN, Piel J. 2016. Biosynthesis of polyketides by *trans*-AT polyketide synthases. Nat Prod Rep 33:231–316. doi:10.1039/c5np00125k. 26689670

[6] Medema MH, Blin K, Cimermancic P, de Jager V, Zakrzewski P, Fischbach MA, Weber T, Takano E, Breitling R. 2011. antiSMASH: rapid identification, annotation and analysis of secondary metabolite biosynthesis gene clusters in bacterial and fungal genome sequences. Nucleic Acids Res 39:W339–46. doi:10.1093/nar/gkr466. 21672958 PMC3125804

[7] Piel J. 2010. Biosynthesis of polyketides by *trans*-AT polyketide synthases. Nat Prod Rep 27:996–1047. doi:10.1039/b816430b. 20464003

[8] Helfrich EJN, Ueoka R, Dolev A, Rust M, Meoded RA, Bhushan A, Califano G, Costa R, Gugger M, Steinbeck C, Moreno P, Piel J. 2019. Automated structure prediction of *trans*-acyltransferase polyketide synthase products. Nat Chem Biol 15:813–821. doi:10.1038/s41589-019-0313-7. 31308532 PMC6642696

[9] Nguyen T, Ishida K, Jenke-Kodama H, Dittmann E, Gurgui C, Hochmuth T, Taudien S, Platzer M, Hertweck C, Piel J. 2008. Exploiting the mosaic structure of *trans*-acyltransferase polyketide synthases for natural product discovery and pathway dissection. Nat Biotechnol 26:225–233. doi:10.1038/nbt1379. 18223641

[10] Jenner M, Afonso JP, Bailey HR, Frank S, Kampa A, Piel J, Oldham NJ. 2015. Acyl-chain elongation drives ketosynthase substrate selectivity in *trans*-acyltransferase polyketide synthases. Angew Chem Int Ed Engl 54:1817–1821. doi:10.1002/anie.201410219. 25529827

[11] Moldenhauer J, Chen X-H, Borriss R, Piel J. 2007. Biosynthesis of the antibiotic bacillaene, the product of a giant polyketide synthase complex of the *trans*-AT family. Angew Chem Int Ed Engl 46:8195–8197. doi:10.1002/anie.200703386. 17886826

[12] Helfrich EJN, Ueoka R, Chevrette MG, Hemmerling F, Lu X, Leopold-Messer S, Minas HA, Burch AY, Lindow SE, Piel J, Medema MH. 2021. Evolution of combinatorial diversity in *trans*-acyltransferase polyketide synthase assembly lines across bacteria. Nature Commun 12:1422. doi:10.1038/s41467-021-21163-x. 33658492 PMC7930024

[13] Blin K, Shaw S, Kloosterman AM, Charlop-Powers Z, van Wezel GP, Medema MH, Weber T. 2021. antiSMASH 6.0: improving cluster detection and comparison capabilities. Nucleic Acids Res 49:W29–W35. doi:10.1093/nar/gkab335. 33978755 PMC8262755

[14] Schleissner C, Cañedo LM, Rodríguez P, Crespo C, Zúñiga P, Peñalver A, La Calle F, Cuevas C. 2017. Bacterial production of a pederin analogue by a free-living marine alphaproteobacterium. J Nat Prod 80:2170–2173. doi:10.1021/acs.jnatprod.7b00408. 28696720

[15] Helfrich EJN, Vogel CM, Ueoka R, Schafer M, Ryffel F, Muller DB, Probst S, Kreuzer M, Piel J, Vorholt JA. 2018. Bipartite interactions, antibiotic production and biosynthetic potential of the Arabidopsis leaf microbiome. Nat Microbiol 3:909–919. doi:10.1038/s41564-018-0200-0. 30038309 PMC7115891

[16] Powell RG, Smith CR, Weisleder D, Matsumoto G, Clardy J, Kozlowski J. 1983. Sesbanimide, a potent antitumor substance from *Sesbania drummondii* seed. J Am Chem Soc 105:3739–3741. doi:10.1021/ja00349a081.

[17] Kačar D, Cañedo LM, Rodríguez P, González EG, Galán B, Schleissner C, Leopold-Messer S, Piel J, Cuevas C, La Calle F, García JL. 2021. Identification of *trans*-AT polyketide clusters in two marine bacteria reveals cryptic similarities between distinct symbiosis factors. Environ Microbiol 23:2509–2521. doi:10.1111/1462-2920.15470. 33734547

[18] Ueoka R, Bortfeld-Miller M, Morinaka BI, Vorholt JA, Piel J. 2018. Toblerols: cyclopropanol-containing polyketide modulators of antibiosis in methylobacteria. Angew Chem 130:989–993. doi:10.1002/ange.201709056. 29112783

[19] Reisberg SH, Gao Y, Walker AS, Helfrich EJN, Clardy J, Baran PS. 2020. Total synthesis reveals atypical atropisomerism in a small-molecule natural product, tryptorubin A. Science 367:458–463. doi:10.1126/science.aay9981. 31896661 PMC6996792

[20] Wyche TP, Ruzzini AC, Schwab L, Currie CR, Clardy J. 2017. Tryptorubin A: a polycyclic peptide from a fungus-derived streptomycete. J Am Chem Soc 139:12899–12902. doi:10.1021/jacs.7b06176. 28853867 PMC5609116

[21] Tietz JI, Schwalen CJ, Patel PS, Maxson T, Blair PM, Tai HC, Zakai UI, Mitchell DA. 2017. A new genome-mining tool redefines the lasso peptide biosynthetic landscape. Nat Chem Biol 13:470–478. doi:10.1038/nchembio.2319. 28244986 PMC5391289

[22] Santos-Aberturas J, Chandra G, Frattaruolo L, Lacret R, Pham TH, Vior NM, Eyles TH, Truman AW. 2019. Uncovering the unexplored diversity of thioamidated ribosomal peptides in Actinobacteria using the RiPPER genome mining tool. Nucleic Acids Res 47:4624–4637. doi:10.1093/nar/gkz192. 30916321 PMC6511847

[23] Helfrich EJN, Lin G-M, Voigt CA, Clardy J. 2019. Bacterial terpene biosynthesis: challenges and opportunities for pathway engineering. Beilstein J Org Chem 15:2889–2906. doi:10.3762/bjoc.15.283. 31839835 PMC6902898

[24] Hannigan GD, Prihoda D, Palicka A, Soukup J, Klempir O, Rampula L, Durcak J, Wurst M, Kotowski J, Chang D, Wang R, Piizzi G, Temesi G, Hazuda DJ, Woelk CH, Bitton DA. 2019. A deep learning genome-mining strategy for biosynthetic gene cluster prediction. Nucleic Acids Res 47:e110. doi:10.1093/nar/gkz654. 31400112 PMC6765103

[25] Cimermancic P, Medema MH, Claesen J, Kurita K, Wieland Brown LC, Mavrommatis K, Pati A, Godfrey PA, Koehrsen M, Clardy J, Birren BW, Takano E, Sali A, Linington RG, Fischbach MA. 2014. Insights into secondary metabolism from a global analysis of prokaryotic biosynthetic gene clusters. Cell 158:412–421. doi:10.1016/j.cell.2014.06.034. 25036635 PMC4123684

